# Association of Coffee Consumption and Prediagnostic Caffeine Metabolites With Incident Parkinson Disease in a Population-Based Cohort

**DOI:** 10.1212/WNL.0000000000209201

**Published:** 2024-03-21

**Authors:** Yujia Zhao, Yunjia Lai, Hilde Konijnenberg, José María Huerta, Ana Vinagre-Aragon, Jara Anna Sabin, Johnni Hansen, Dafina Petrova, Carlotta Sacerdote, Raul Zamora-Ros, Valeria Pala, Alicia K. Heath, Salvatore Panico, Marcela Guevara, Giovanna Masala, Christina M. Lill, Gary W. Miller, Susan Peters, Roel Vermeulen

**Affiliations:** From the Institute for Risk Assessment Sciences (Y.Z., H.K., S. Peters, R.V.), Utrecht University, the Netherlands; Department of Environmental Health Sciences (Y.L., G.W.M.), Mailman School of Public Health, Columbia University, New York, NY; Department of Epidemiology (J.M.H.), Murcia Regional Health Council-IMIB, Murcia; CIBER Epidemiología y Salud Pública (CIBERESP) (J.M.H., M.G.), Madrid; Movement Disorders Unit (A.V.-A.), Department of Neurology, University Hospital Donostia; BioDonostia Health Research Institute (A.V.-A.), Neurodegenerative Diseases Area, San Sebastián, Spain; Division of Cancer Epidemiology (J.A.S.), German Cancer Research Center (DKFZ), Heidelberg, Germany; Danish Cancer Institute (J.H.), Danish Cancer Society, Copenhagen, Denmark; Escuela Andaluza de Salud Pública (EASP) (D.P.); Instituto de Investigación Biosanitaria-ibs.GRANADA (D.P.), Granada; Centro de Investigación Biomédica en Red de Epidemiología y Salud Pública (CIBERESP) (D.P.), Madrid, Spain; Unit of Cancer Epidemiology (C.S.), Città della Salute e della Scienza University-Hospital, Turin, Italy; Unit of Nutrition and Cancer (R.Z.-R.), Cancer Epidemiology Research Programme, Catalan Institute of Oncology (ICO), Bellvitge Biomedical Research Institute (IDIBELL), Barcelona, Spain; Epidemiology and Prevention Unit (V.P.), Fondazione IRCCS Istituto Nazionale dei Tumori di Milano, Italy; Department of Epidemiology and Biostatistics (A.K.H., M.G.), School of Public Health, Imperial College London, United Kingdom; School of Medicine (S. Panico), Federico II University, Naples, Italy; de Salud Pública y Laboral de Navarra (M.G.), Pamplona; Navarra Institute for Health Research (IdiSNA) (M.G.), Pamplona, Spain; Institute for Cancer Research (G.M.), Prevention and Clinical Network (ISPRO), Florence, Italy; Institute of Epidemiology and Social Medicine (C.M.L.), University of Münster, Germany; Ageing Epidemiology Research Unit (AGE) (C.M.L.), School of Public Health, Imperial College London, United Kingdom; and University Medical Centre Utrecht (R.V.), the Netherlands.

## Abstract

**Background and Objectives:**

Inverse associations between caffeine intake and Parkinson disease (PD) have been frequently implicated in human studies. However, no studies have quantified biomarkers of caffeine intake years before PD onset and investigated whether and which caffeine metabolites are related to PD.

**Methods:**

Associations between self-reported total coffee consumption and future PD risk were examined in the EPIC4PD study, a prospective population-based cohort including 6 European countries. Cases with PD were identified through medical records and reviewed by expert neurologists. Hazard ratios (HRs) and 95% CIs for coffee consumption and PD incidence were estimated using Cox proportional hazards models. A case-control study nested within the EPIC4PD was conducted, recruiting cases with incident PD and matching each case with a control by age, sex, study center, and fasting status at blood collection. Caffeine metabolites were quantified by high-resolution mass spectrometry in baseline collected plasma samples. Using conditional logistic regression models, odds ratios (ORs) and 95% CIs were estimated for caffeine metabolites and PD risk.

**Results:**

In the EPIC4PD cohort (comprising 184,024 individuals), the multivariable-adjusted HR comparing the highest coffee intake with nonconsumers was 0.63 (95% CI 0.46–0.88, *p* = 0.006). In the nested case-control study, which included 351 cases with incident PD and 351 matched controls, prediagnostic caffeine and its primary metabolites, paraxanthine and theophylline, were inversely associated with PD risk. The ORs were 0.80 (95% CI 0.67–0.95, *p* = 0.009), 0.82 (95% CI 0.69–0.96, *p* = 0.015), and 0.78 (95% CI 0.65–0.93, *p* = 0.005), respectively. Adjusting for smoking and alcohol consumption did not substantially change these results.

**Discussion:**

This study demonstrates that the neuroprotection of coffee on PD is attributed to caffeine and its metabolites by detailed quantification of plasma caffeine and its metabolites years before diagnosis.

## Introduction

Parkinson disease (PD) is the most common motor neurodegenerative disorder for which there is no effective prevention or curative treatment available so far. Coffee consumption has been associated with a reduced risk of PD in several prospective cohorts during the past 20 years.^1-5^ The protective effect was also present for caffeine from noncoffee sources, such as tea, cola beverages, and chocolate.^2-4^ By contrast, the effect was not observed for decaffeinated coffee,^5^ suggesting that the inverse association between coffee consumption and PD is largely due to caffeine and its metabolites, rather than other bioactive compounds in coffee. However, these findings were based on food questionnaire data rather than on measuring caffeine or its metabolites in predisease biological samples.

Some exploratory case-control studies have indicated that blood concentrations of caffeine and its major metabolites in humans, namely paraxanthine and theophylline, were reduced in patients with prevalent PD when compared with healthy individuals.^6-8^ Following these observations, clinical trials have been initiated to investigate whether caffeine or its metabolites could slow the progression of PD. Unfortunately, these studies have shown no benefit of caffeine and its metabolites on symptom attenuation and progression in PD.^9,10^ However, no studies to date have prospectively investigated the role of caffeine levels in prediagnostic samples to investigate whether caffeine and its metabolites could be protective in a prodromal state of the disease. This research question can only be investigated in very large cohorts with baseline blood samples and long follow-up available, such as in the European Prospective Investigation into Cancer and Nutrition (EPIC) cohort. The EPIC cohort comprises more than half million participants across Europe who have been followed up for >20 years and for which baseline blood samples were collected and ascertained in a highly standardized fashion.^11^ During the long follow-up, several hundred participants have been diagnosed with PD.^12^

Coffee is the most widely consumed psychoactive beverage in the world. Unraveling the biological action of caffeine on PD not only carries important public health implications but also enhances our understanding of PD etiology and fosters potential prevention strategies. In this study, we aimed to investigate the relationship between caffeine and future PD risk prospectively in the EPIC cohort, using self-reported coffee consumption and direct measurement of prediagnostic caffeine and its metabolites.

## Methods

### The EPIC4PD Cohort

The EPIC is an ongoing prospective cohort study designed to explore the relationship between nutrition and noncommunicable diseases.^11^ Baseline recruitment was conducted between 1992 and 2000 across 23 centers in 10 European countries. The EPIC cohort comprises 519,978 participants (366,521 women and 153,457 men), mostly aged 35–70 years at recruitment. At enrollment, comprehensive dietary habits and lifestyle data were collected using questionnaires. Moreover, anthropometric measurements were conducted, and blood samples were obtained.^11^

To prospectively investigate the association between prediagnostic risk factors and the incidence of PD, a substudy known as EPIC4PD was initiated with the EPIC cohort.^12^ The inclusion of study centers in EPIC4PD depended on the availability of neurologists for PD ascertainment. Ultimately, the EPIC4PD was based on a source population of 192,980 individuals from 6 countries, including Sweden (Umeå and Malmö), the United Kingdom (Cambridge), the Netherlands (Utrecht), Germany (Heidelberg), Spain (Navarra, San Sebastián, and Murcia), and Italy (Turin, Varese, Florence, and Naples). The Naples and Utrecht cohorts exclusively comprised women, while all the other cohorts included participants of both sexes. To date, follow-up for the EPIC4PD is 98.5% complete, and the median duration of follow-up of the entire population is 12.8 years (maximum 20.8 years).^12^

### Case Ascertainment and Study Population

In brief, cases with potential PD were identified through record linkage and validated by experts in movement disorders through clinical records.^12^ Reliability of diagnoses was determined by the quality of clinical data (rated as “poor,” “good,” or “excellent”) and the confidence degree of the neurologist expert on the basis of their final judgment (rated as “low,” “medium,” or “high”). Diagnoses were defined as “definite” only when the confidence degree of the neurologist was high and the data quality was excellent; “very likely” when the confidence degree was high, while data quality was either good or poor; “probable” when the confidence degree was medium and data quality was either excellent or good; and “possible” in all remaining cases. A total of 786 cases with PD was ascertained. Cases who received a diagnosis after the date of recruitment were defined as incident cases (n = 639).^12^

Our study consisted of 2 parts, including a prospective cohort study (EPIC4PD) and a nested case-control study, to interrogate the links of coffee consumption and caffeine and its metabolites with PD risk, respectively. For the EPIC4PD, several exclusion criteria were applied: cases with prevalent PD and cases without date of diagnosis (n = 147); participants with PD-like conditions (multiple system atrophy, progressive supranuclear palsy, vascular parkinsonism, dementia with Lewy bodies, essential tremor, PD with essential tremor, and unclassifiable parkinsonism) (n = 214); those with missing information on coffee consumption and smoking status at recruitment (n = 8,484); and those with extreme coffee consumption (>2,500 mL/d) (n = 111), to exclude possible bias related to caffeine addiction.

Cases with incident PD within the EPIC4PD study were considered for inclusion in the nested case-control study, provided that a plasma sample was accessible. Individuals from Sweden were excluded due to the unavailability of plasma samples. For each case with PD, 1 control was selected by incidence density sampling matched for age at recruitment, sex, study center, and fasting status at blood collection.

### Dietary and Lifestyle Data

Dietary intake was assessed by a dietary questionnaire that had been developed and validated in each participating country. A face-to-face dietary interview was applied in Spain, while self-administered questionnaires were used in other countries.^11^ To increase comparability across the study centers, a standardized 24-hour diet recall was collected, as a reference calibration method, from a stratified random sample of 36,900 individuals from the entire EPIC cohort.^13^ Total coffee consumption was available for all countries. Caffeinated coffee consumption was available for almost all centers except for Naples and Umeå. Information regarding decaffeinated coffee consumption was collected from participants in Germany, Italy (excluding Naples), the Netherlands, and the United Kingdom.^14^ Participants reported the number of cups of coffee consumed per month, week, or day. Daily coffee consumption (in milliliters) was then calculated using the typical sizes of cups for each center.^14^

Participants also completed questionnaires on lifestyle including smoking and alcohol consumption, education level, and physical activity. Female participants additionally reported menopausal status and hormone usage. Height and weight were measured following standardized protocols, and body mass index (BMI) was subsequently calculated.^11^

### Caffeine Metabolite Measurement

In the nested case-control study, plasma samples for the participants were sourced from the cohort biobank at the International Agency for Research on Cancer (IARC). These samples were collected between 1992 and 1998, with on average 8 years before the diagnosis of PD in cases. To profile circulating caffeine metabolites in plasma, we performed untargeted metabolomics analysis using a liquid chromatography-high resolution mass spectrometry–based platform as previously described^15,16^ (eAppendix 1, links.lww.com/WNL/D497). To maximize the detection of polar and nonpolar metabolites, 2 complementary analyses were performed, namely hydrophilic interaction liquid chromatography-electrospray ionization (ESI)(+) and reverse-phase chromatography-ESI(−), termed as “HILpos” and “C18neg,” both operated in full scan mode at 120,000 mass resolution with a mass-to-charge ratio (m/z) range of 85–1,275. Raw data files were extracted and aligned using apLCMS R package^17^ and further processed through xMSanalyzer^18^ and corrected for batch effects (ComBat). Uniquely detected peaks consisting of m/z, retention time (RT), and ion abundance were referred to as metabolite features. In total, 9,435 features for HILpos and 8,439 for C18neg were yielded.

Structural annotation of compounds of caffeine metabolism was implemented through an integrated cheminformatic strategy. We retrieved a complete set of 22 structures from caffeine metabolism referencing the Kyoto Encyclopedia of Genes and Genomes,^19^ alongside 1,3,7-trimethyldihydrourate, a novel caffeine metabolite recently discovered through our in vitro exposomic platform.^20^ We first built internal RT-m/z libraries for HILpos and C18neg modes, including caffeine and its major metabolites. Meanwhile, to expand the coverage, we leveraged in silico cheminformatic analyses for annotating all plausible metabolites involved in caffeine metabolism. Using accurate m/z, isotopic ratios, and RT, we annotated detected peaks based on formula prediction^21,22^ and RT estimation by XGBoost algorithm^23^ and manually curated based on extensive bioanalytical inferences and expert consultation. Annotation confidence was assigned as level 1 for features matched with our in-house library and level 2 for features with predicted parameters but not validated by authentic chemical standards.^24^

A total of 15 features were successfully annotated, corresponding to 12 unique caffeine metabolites. Three metabolites, 5-acetylamino-6-amino-3-methyluracil (AAMU), 1-methylxanthine, and 3,6,8-trimethylallantoin, were detected in both HILpos and C18neg modes.

### Statistical Analysis

In the EPIC4PD, coffee consumers were binned into quartiles based on the distribution in each country (country-specific quartiles), to account for heterogeneity of consumed volume and concentration of coffee between countries.^14^ Hazard ratios (HRs) and their corresponding 95% CIs for PD risk were estimated using Cox proportional hazards models, with age as the underlying time variable. The entry time for all participants was defined as age at recruitment, and the exit time was either the age at diagnosis for cases with PD or the last date when follow-up was deemed complete for participants without PD. We also performed analysis using coffee overall, noncountry-specific quartiles (based on data from all countries combined). Exposure-response effect of coffee intake on PD was examined by entering the categorical value of the quartiles (0 for nonconsumers and 1–4 for coffee quartiles) into the model as a continuous term. PD risk was also estimated per 100 mL/d coffee intake. To assess the impact of coffee consumption in the population, the population preventable fraction (PPF) was calculated. PPF is defined as the proportion of cases with PD that could be prevented within the population if coffee intake were intervened upon (formula in eAppendix 2, links.lww.com/WNL/D497).

Age, male sex, and smoking are well-recognized risk factors of PD,^25,26^ and they might influence coffee-consumption habits. Moreover, there might be systematic differences in data collection among study centers. Thus, the main analyses were adjusted for age at recruitment, sex, study center, and smoking status at recruitment (never, former, and current smoker). In additional analyses, a set of confounders were also considered, including BMI, alcohol consumption (nonconsumer, 0.1–5, 5–15, 15–30, 30–60, and ≥60 g/d), physical activity (inactive, moderately inactive, moderately active, and active and not specified), and education level (none, primary school, secondary/technical school, longer education, and not specified). Age (in years) and BMI (in kg/m^2^) were included in the Cox models as continuous variables, while categorical variables were represented using dummy codes in the models (“male” and “Italy” as reference for sex and study center, respectively; lowest level as reference for alcohol consumption, physical activity, and education level). None of the variables in additional analyses considerably modified the risk estimates (eTable 1, links.lww.com/WNL/D497), and they were thus not included in the final models.

The main analyses were stratified by sex and smoking status to account for possible effect modifications. In the subgroup analysis of women, menopausal status (premenopausal, postmenopausal, perimenopausal, and ovariectomy) and history of using hormone therapy (ever used or not) were further adjusted. Possible interactions between sex or smoking status and coffee intake were tested using the likelihood ratio test based on models with and without the interaction terms.

Heterogeneity across countries was investigated using a meta-analytic approach based on HRs of coffee consumers compared with nonconsumers in each country. The *I*^2^ statistic was used to illustrate the proportion of observed variance that reflects true variance among countries rather than sampling error.^27^ *I*^2^ values of 25%, 50% ,and 75% represent low, moderate, and high levels of heterogeneity. Sensitivity analyses were performed limiting to “definite” and “very likely” cases with PD (n = 314). To rule out possible reverse causality, our analyses were limited to cases with PD diagnosed after 8 years (median) since recruitment into the cohort. In addition, we further conducted analyses after exclusion of cases by consecutive 1-year interval of prediagnostic periods (from >0 to >16 years).

To account for the potential role of caffeine in the effects of coffee on PD, stratified analyses were conducted for caffeinated and decaffeinated coffee consumption. Participants for whom the sum of both coffee subtypes was equal to the total coffee intake were included in stratified analyses. Caffeinated and decaffeinated coffee consumers were divided into country-specific tertiles due to the smaller sample size, and models for caffeinated and decaffeinated coffee were mutually adjusted for one another. Coffee consumers were additionally categorized according to coffee types they consumed (only caffeinated, only decaffeinated, and both types of coffee).

In the nested case-control study, missing values of the detected caffeine metabolites (missing percentage ranging between 0% and 64.1%, eFigure 1, links.lww.com/WNL/D497) that were below limits of detection were imputed using a quantile regression approach for left-censored missing data based on distributions of available values of metabolites, as implemented in imputeLCMD R package.^28^ Correlations among the metabolites and correlations between coffee consumption volume and metabolites were examined by Spearman correlation (ρ). Ion intensities of metabolites were log2 transformed to reduce influence of extreme values and scaled (divided by SD) to make results of analysis comparable. Conditional logistic regression for the matched case-control sets was applied to estimate odds ratios (ORs) and 95% CIs for associations between caffeine metabolites and PD, adjusting for smoking status. The nested case-control study adopted the same stratified and sensitivity analyses as in the analysis of coffee consumption and PD in the cohort to evaluate the robustness of results.

### Standard Protocol Approvals, Registrations, and Patient Consents

The EPIC study was approved by the ethical committee of the IARC and by the ethical review boards of each study center. All participants provided written informed consent.

### Data Availability

The datasets used and analyzed in this study are not publicly available due to privacy agreements.

## Results

### Study Population

Following the application of exclusion criteria, our analysis included a total of 184,024 participants from the EPIC4PD cohort, with a median follow-up of 13.1 years. Within this cohort, 308 and 285 cases with incident PD were recorded among men and women, respectively (Table 1). The age-adjusted incidence rates for individuals aged 65 years and older were 134 and 77 per 100,000 person-years for men and women, respectively. The median period between recruitment and PD diagnosis was 8.3 years. The median age at recruitment for individuals with PD was higher than those without PD (61.2 vs 52.6 years). The prevalence of coffee consumption in the entire EPIC4PD population was 93%. The daily coffee consumption volume was highest in the Netherlands (median 500 mL/d) and lowest in Italy and Spain (median 100 mL/d for both countries) (eTable 2, links.lww.com/WNL/D497). Participants in the highest quartile of coffee intake were more likely to be men, current smokers, and younger and reported higher alcohol consumption (eTable 3). In our nested case-control study, which included 351 cases with incident PD and 351 matched controls, the demographics, lifestyle factors, and coffee consumption were comparable with those observed in the EPIC4PD cohort (Table 1).

**Table 1:** Baseline Characteristics Among Participants in the EPIC4PD Cohort and Nested Case-Control Study

Characteristic	EPIC4PD cohort (n = 184,024)		Nested case-control study (n = 702)^a^	
	Cases with PD (n = 593)	Noncases (n = 183,431)	Cases with PD (n = 351)	Controls (n = 351)
Age at recruitment (y), median (IQR)	61.2 (55.2–65.8)	52.6 (46.7–59.9)	60.7 (54.8–65.6)	60.4 (55.0–65.2)
Age at diagnosis (y), median (IQR)	69.8 (63.6–74.4)	—	68.7 (62.8–74.0)	—
Years between recruitment and diagnosis, median (IQR)	8.3 (4.9–11.5)	—	7.8 (4.6–11.0)	—
Definite and very likely cases, n (%)	314 (53)	—	188 (54)	—
Sex, n (%)				
Male	308 (52)	67,442 (37)	195 (56)	195 (56)
Female	285 (48)	115,989 (63)	156 (44)	156 (44)
Country^b^				
Italy	64 (11)	40,111 (22)	54 (15)	54 (15)
Spain	101 (17)	24,852 (13)	97 (28)	97 (28)
United Kingdom	170 (29)	23,227 (13)	141 (40)	141 (40)
Netherlands	13 (2)	16,813 (9)	13 (4)	13 (4)
Germany	50 (8)	25,349 (14)	46 (13)	46 (13)
Sweden	195 (33)	53,079 (29)	—	—
Coffee consumption at recruitment (mL/d)				
Nonconsumer, n (%)^c^	67 (11)	12,826 (7)	45 (12.9)	36 (10.4)
Total coffee, median (IQR)^c^	261 (104–475)	286 (113–500)	190 (79–475)	190 (73–475)
Caffeinated coffee, median (IQR)^d^	261 (100–475)	261 (90–475)	190 (60–475)	190 (82–475)
Decaffeinated coffee, median (IQR)^e^	190 (47–332)	62 (25–190)	190 (48–300)	86 (20–273)
Smoking status at recruitment, n (%)^f^				
Never smoker	321 (54)	85,717 (47)	183 (52)	174 (49)
Former smoker	198 (33)	53,557 (29)	115 (33)	109 (31)
Current smoker	74 (13)	44,157 (24)	43 (12)	55 (16)
BMI at recruitment (kg/m^2^), median (IQR)	25.8 (23.8–28.5)	25.4 (23.1–28.2)	26.5 (24.2–29.2)	26.0 (23.8–29.1)
Alcohol consumption at recruitment (g/d)^g^				
Nonconsumer, n (%)	118 (20)	30,994 (17)	77 (22)	62 (18)
Total alcohol, median (IQR)	7.4 (2.7–18.7)	7.4 (2.1–18.8)	9.1 (2.8–24.2)	10.6 (2.6–26.7)
Higher education, n (%)^h^	84 (14)	32,625 (18)	41 (12)	46 (13)
Physically active, n (%)^i^	24 (4)	15,674 (9)	18 (5)	26 (7)
Postmenopausal, n (%)^j^	220 (77)	57,280 (49)	—	—
Ever use of menopausal hormone therapy, n (%)^j,k^	59 (21)	24,388 (21)	—	—

Abbreviations: BMI = body mass index; EPIC = European Prospective Investigation into Cancer and Nutrition; IQR = interquartile range; PD = Parkinson disease.

a Cases with PD and controls were matched on age at recruitment, sex, country, and fasting status in the nested case-control study.

b No individuals from Sweden were included in the nested case-control study.

c Information on total coffee was missing for 3 cases with PD and 4 controls in the nested case-control study.

d Information on caffeinated coffee was missing for 68 cases with PD and 30,359 participants without PD in the EPIC4PD cohort and for 6 cases with PD and 7 controls in the nested case-control study.

e Information on decaffeinated coffee was missing for 309 cases with PD and 82,974 participants without PD in the EPIC4PD cohort and for 103 cases with PD and 104 controls in the nested case-control study.

f Information on smoking status was missing for 10 cases with PD and 13 controls in the nested case-control study.

g Information on alcohol consumption was missing for 3 cases with PD and 4 controls in the nested case-control study.

h Information on education level was missing for 20 cases with PD and 1,729 participants without PD in the EPIC4PD cohort and for 24 cases with PD and 17 controls in the nested case-control study.

i Information on physical activity was missing for 58 cases with PD and 25,590 participants without PD in the EPIC4PD cohort and for 13 cases with PD and 10 controls in the nested case-control study.

j Only among women.

k Information on ever use of menopausal hormone therapy was missing for 55 cases with PD and 16,667 participants without PD in the EPIC4PD cohort.

### Coffee Consumption and PD

An inverse exposure-response relationship between coffee consumption and PD was observed (*p* = 0.003) with an HR of 0.63 (95% CI 0.46–0.88) for the highest quartile of consumers vs nonconsumers (Table 2). HRs based on overall coffee intake quartiles were similar to those using country-specific quartiles (eTable 4, links.lww.com/WNL/D497). The point estimates of HR for coffee consumers compared with nonconsumers varied between 0.37 and 0.95 across countries, with a minimal heterogeneity noted (*I*^2^ = 3.3%) (eFigure 2). The PPF, with the HR of coffee consumers vs nonconsumers at 0.72 (95% 0.56–0.94), was 26% (95% CI 6.6%–41%) for coffee consumption in the EPIC4PD population. In subanalyses, the inverse association was limited to caffeinated coffee consumers (HR for highest tertile vs nonconsumers 0.57, 95% CI 0.35–0.94; *p* = 0.007), and no association was observed for decaffeinated coffee consumption (eTable 5).

**Table 2:** Associations of Total Coffee Consumption and Risk of PD in the EPIC4PD Cohort

Analysis	Coffee consumption^a^					*p* for trend	Per 100 mL/d
	Nonconsumers	Quartile 1	Quartile 2	Quartile 3	Quartile 4		
All participants (n = 184,024)							
Cases with PD, n	67	203	145	90	88		
HR (95% CI)^b^	Reference	0.80 (0.61–1.06)	0.71 (0.53–0.96)	0.66 (0.48–0.91)	0.63 (0.46–0.88)	0.003	0.97 (0.94–1.00)
Men (n = 67,750)							
Cases with PD, n	36	105	74	45	48		
HR (95% CI)^b^	Reference	0.81 (0.56–1.19)	0.68 (0.46–1.02)	0.70 (0.45–1.11)	0.69 (0.44–1.07)	0.090	0.97 (0.93–1.01)
Women (n = 116,274)							
Cases with PD, n	31	98	71	45	40		
HR (95% CI)^b^	Reference	0.78 (0.52–1.17)	0.75 (0.49–1.15)	0.63 (0.39–1.00)	0.60 (0.37–0.96)	0.025	0.98 (0.93–1.03)
Never smokers (n = 86,038)							
Cases with PD, n	41	103	83	55	39		
HR (95% CI)^b^	Reference	0.68 (0.47–0.97)	0.72 (0.49–1.04)	0.69 (0.46–1.05)	0.59 (0.38–0.93)	0.107	1.00 (0.95–1.05)
Former smokers (n = 53,755)							
Cases with PD, n	99		41	24	34		
HR (95% CI)^b^	Reference^c^		0.64 (0.45–0.92)	0.63 (0.39–0.99)	0.78 (0.52–1.16)	0.085	0.96 (0.91–1.01)
Current smokers (n = 44,231)							
Cases with PD, n	27		21	11	15		
HR (95% CI)^b^	Reference^c^		0.97 (0.55–1.72)	0.62 (0.30–1.30)	0.67 (0.35–1.28)	0.117	0.95 (0.87–1.04)
Late-diagnosed cases^d^ (n = 183,743)							
Cases with PD, n	38	110	69	47	48		
HR (95% CI)^b^	Reference	0.77 (0.53–1.11)	0.60 (0.41–0.90)	0.50 (0.32–0.77)	0.54 (0.35–0.84)	0.001	0.96 (0.91–1.01)
Definite and very likely cases (n = 183,745)							
Cases with PD, n	33	95	90	52	44		
HR (95% CI)^b^	Reference	0.80 (0.54–1.19)	0.90 (0.60–1.34)	0.72 (0.46–1.13)	0.62 (0.39–0.98)	0.050	0.97 (0.93–1.02)

Abbreviations: EPIC = European Prospective Investigation into Cancer and Nutrition; HR = hazard ratio; PD = Parkinson disease.

a Based on country-specific quartiles for coffee consumers. Quartile cutoffs were 62, 100, and 145 mL/d in Italy, 47, 100, and 184 mL/d in Spain, 190, 475, and 557 mL/d in the United Kingdom, 375, 500, and 750 mL/d in the Netherlands, 261, 392, and 573 mL/d in Germany, and 300, 400, and 601 mL/d for Sweden.

b Cox regression adjusted for age at recruitment, sex (when appropriated), country, and smoking status (when appropriated).

c Reference category merged with quartile 1 due to low case numbers among nonconsumers.

d Cases with PD diagnosed within 8 years of follow-up were excluded.

No obvious difference was noted for associations between men and women (*p* for interaction = 0.974), although a statistically significant trend for coffee intake and PD was only found in women (*p* = 0.025) (Table 2). Further adjustment for menopause status and hormone use did not materially change the associations among women (eTable 6, links.lww.com/WNL/D497). A slightly stronger association for the highest quartile was observed among hormone never users (HR 0.50, 95% CI 0.26–0.96).

A stronger association between coffee consumption and PD was observed in never smokers (HR for highest quartile vs nonconsumers 0.59, 95% CI 0.38–0.93) than in former and current smokers (Table 2). Interaction between smoking and coffee intake was not significant (*p* for interaction = 0.185). Furthermore, compared with individual effect of smoking and coffee, a more pronounced inverse association was observed for participants who were both cigarette smokers and coffee drinkers at baseline (HR vs nonconsumers for both cigarettes and coffee 0.41, 95% CI 0.29–0.59) (eTable 7, links.lww.com/WNL/D497).

In the analyses limited to 281 cases with PD diagnosed after 8 years of follow-up, the associations between coffee intake and PD were strengthened across all quartiles (HR for highest quartile 0.54, 95% CI 0.35–0.84) (Table 2). Slightly stronger inverse associations with increasing prediagnostic time lags were also reflected when we progressively excluded cases diagnosed within a certain time frame (eFigure 3, links.lww.com/WNL/D497). Estimates based on the analysis limiting to 314 definite and very likely cases were similar to those in the main analysis (Table 2).

### Caffeine Metabolites and PD

Prediagnostic levels of most caffeine metabolites were positively associated with self-reported coffee volume, as indicated by correlation coefficients ranging from 0.10 to 0.41 (Table 3). Several metabolites including caffeine, theophylline, paraxanthine, and AAMU (C18neg) were moderately correlated with each other (correlation coefficient, ρ >0.4) (eFigure 4, links.lww.com/WNL/D497).

**Table 3:** Associations of Caffeine Metabolites and Parkinson Disease Risk in the Nested Case-Control Study (n = 702)

LC-MS mode	Retention time (s)	m/z	Metabolite name	Annotation level^a^	Correlation with coffee^b^	OR (95% CI)^c^ Per SD increase of log2 ion intensity
HILIC-positive	195.0877	30.5	Caffeine	1	0.41	0.80 (0.67–0.95)
HILIC-positive	181.0773	31.8	Paraxanthine	2	0.21	0.82 (0.69–0.96)
HILIC-positive	181.0720	32.9	Theophylline	1	0.41	0.78 (0.65–0.93)
HILIC-positive	211.0770	57.2	1,3,7-Trimethyluric acid	2	−0.10	0.92 (0.77–1.10)
HILIC-positive	227.0789	39.5	AFMU	1	−0.16	1.19 (0.99–1.43)
HILIC-positive	199.0821	74.8	AAMU (HILpos)	2	0.12	1.05 (0.89–1.23)
C18-negative	197.0681	31.8	AAMU (C18neg)	1	0.42	0.94 (0.79–1.11)
HILIC-positive	167.0562	31.4	1-Methylxanthine (HILpos)	1	0.41	0.93 (0.78–1.09)
C18-negative	165.0414	33.0	1-Methylxanthine (C18neg)	1	0.44	0.87 (0.73–1.04)
HILIC-positive	153.0408	42.3	Xanthine	1	0.10	1.01 (0.86–1.20)
HILIC-positive	183.0505	69.9	1-Methyluric acid	2	0.13	0.84 (0.72–0.98)
HILIC-positive	201.0892	44.4	3,6,8-Trimethylallantoin (HILpos)	2	0.14	1.00 (0.83–1.21)
C18-negative	199.0782	32.9	3,6,8-Trimethylallantoin (C18neg)	2	−0.08	1.06 (0.90–1.26)
C18-negative	225.0627	33.8	1,3,7-Trimethyl-5-hydroxyisourate	2	−0.16	1.19 (0.99–1.41)
C18-negative	211.0837	33.2	1,3,7-Trimethyldihydrouric acid	2	0.41	0.85 (0.72–1.01)

Abbreviations: AAMU = 5-acetylamino-6-amino-3-methyluracil; AFMU = 5-acetylamino-6-formylamino-3-methyluracil; HILIC = hydrophilic interaction liquid chromatography; LC-MS = liquid chromatography coupled with high-resolution mass spectrometry; m/z = mass/charge; OR = odds ratio

a Features matched with the in-house library were assigned with level 1 annotation and features matched with predicted chemical retention time with level 2 annotation.

b Spearman correlation coefficient between continuous coffee volume (in mL/d) and ion intensity of caffeine metabolites.

c Conditional logistic regression for the matched case-control sets, adjusted for smoking status.

Caffeine and 3 other metabolites (paraxanthine, theophylline, and 1-methyluric acid) were negatively associated with PD risk (OR per SD increase (95% CI) 0.80 (0.67–0.95), 0.82 (0.69–0.96), 0.78 (0.65–0.93), and 0.84 (0.72–0.98), respectively) (Table 3). Subtle associations with PD risk, although not statistically significant, were observed for 1,3,7-trimethyldihydrouric acid (OR 0.85, 95% CI 0.72–1.01), 5-acetylamino-6-formylamino-3-methyluracil (AFMU) (OR 1.19, 95% CI 0.99–1.43), and 1,3,7-trimethyl-5-hydroxyisourate (OR 1.19, 95% CI 0.99–1.41). Analyses for caffeine and theophylline were more pronounced among men than women (*p* for interaction by sex 0.020 and 0.011, respectively) (Figure 1). There was no evidence for effect modification by smoking status for the associations between caffeine metabolites and PD (eFigure 5, links.lww.com/WNL/D497), although the inverse association for caffeine was stronger among current smokers (OR 0.55, 95% CI 0.31–0.92) than in noncurrent smokers. Furthermore, sensitivity analyses of limiting to cases diagnosed after 8 years since recruitment and cases with high validity did not reveal substantial changes (eFigure 6).

**Figure:**
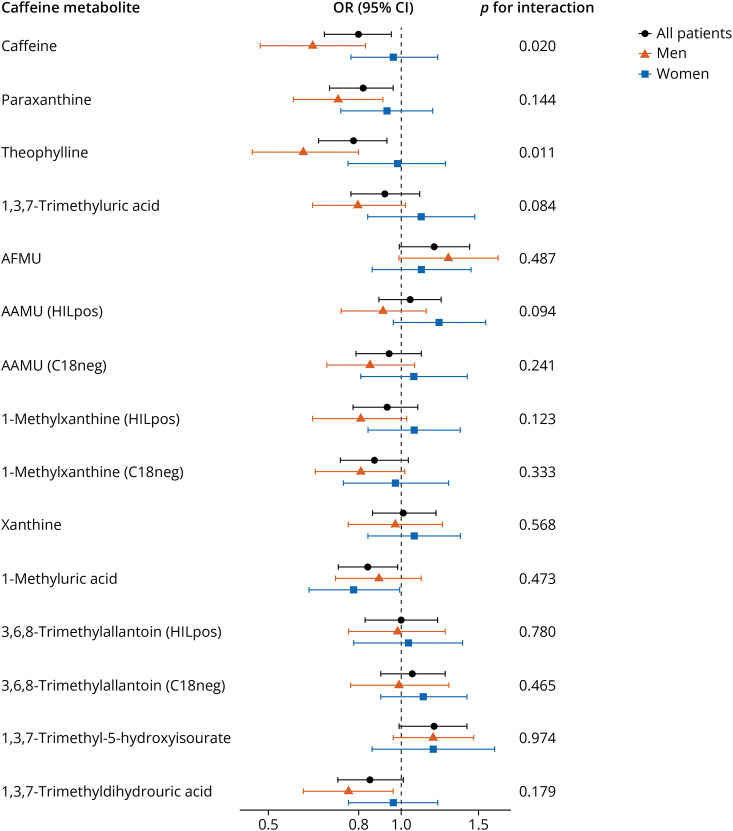
Associations Between Caffeine Metabolites and Parkinson Disease Risk Among Men and Women in the Nested Case-Control Study (n = 702) ORs and CIs (per SD increase of log2 ion intensity) were calculated by conditional logistic regression for the matched case-control sets, adjusted for smoking status, for men and women separately. *p* Values for interaction of sex and metabolite were estimated by likelihood ratio test based on models with and without the interaction terms. AFMU = 5-acetylamino-6-formylamino-3-methyluracil; AAMU = 5-acetylamino-6-amino-3-methyluracil; OR = odds ratio.

Analyses by increasing prediagnostic periods for caffeine metabolites showed that for caffeine and the major metabolites paraxanthine and theophylline, associations became slightly stronger with increasing prediagnostic periods (eFigure 7, links.lww.com/WNL/D497). Of interest, for several downstream metabolites (1-methyluric acid, 1,3,7-trimethyldihydrouric acid, AFMU, and 1,3,7-trimethyl-5-hydroxyisourate), the associations became weaker for prediagnostic periods longer than 10 years.

## Discussion

This study demonstrated an inverse association of caffeinated coffee consumption with the risk of PD in one of the largest longitudinal cohorts worldwide with more than 20 years of follow-up. The neuroprotective effects of coffee were exposure dependent, and individuals in the highest coffee consumption group had nearly 40% lower risk of PD compared with nonconsumers. This observation was strengthened with a comprehensive evaluation of prospectively measured plasma caffeine and its metabolites. These analyses showed strong inverse associations for caffeine and its major metabolites with the risk of PD.

The EPIC4PD population is not a strictly random sample for the entire European population, with women outnumbering men in the cohort. There was still a slight predominance of men among patients with PD. Notably, individuals who developed PD were generally older during recruitment compared with those who did not develop PD. These findings further underscore the important role of aging and male sex for PD risk. Moreover, incidence of PD in the EPIC4PD cohort was comparable with those reported in the North America (162–277 per 100,000 person-years among male participants, 66–161 per 100,000 person-years among female participants).^29^ Several large US prospective cohorts have reported comparable effect sizes for the highest coffee intake group (with adjusted HR ranging from 0.43 to 0.81 for men and from 0.61 to 0.90 for women), which aligns with our findings.^5,30,31^

The strength of our study was using objective blood markers for caffeine metabolism, which largely mitigates regional variations on coffee consumption. More notably, our study largely minimizes the possibility of reverse causation by collecting blood samples before PD diagnosis. By contrast, previous studies analyzed caffeine biochemical markers in biosamples from individuals who had been living with PD, with an average disease duration ranging from 6 to 8 years.^6-8^ This approach introduced potential bias because these patients might change their coffee consumption habits due to smell and taste dysfunction. In addition, caffeine is primarily metabolized by CYP1A2, an isoform of the hepatic cytochrome P450 enzyme family.^32^ Antiparkinsonian drugs such as levodopa has been shown to upregulate CYP1A2 activities, resulting in an increased metabolism of caffeine.^33^

Coffee consumption has long been suggested to reduce or delay the development of PD, with caffeine identified as the most likely causal factor.^1,2,5^ Caffeine administration attenuated motor impairment, neuronal death, and dopamine depletion in various animal models of PD.^34-36^ It is believed that the neuroprotective effects of caffeine are mainly attributed to the blocking of adenosine 2A receptor (A2AR).^37^ Moreover, 2 major caffeine metabolites, paraxanthine and theophylline, have demonstrated the ability to mitigate symptoms in animal models with PD.^38,39^ These neuroprotective effects align with our findings, which revealed an inverse association between caffeine, paraxanthine, and theophylline and the incidence of PD. Of more importance, these associations tended to be marginally stronger with increasing prediagnostic period, indicating minimal effect due to reverse causality.

On the contrary, other caffeine-derived metabolites, specifically AFMU, 1,3,7-trimethyldihydrouric acid, and 1,3,7-trimethyl-5-hydroxyisourate, exhibited altered associations with PD, although these associations did not reach statistical significance. Notably, these associations were absent when the time between biomarker assessment and PD diagnosis was longer than 10 years. The molecular underpinnings of these metabolites remain elusive, partially due to lack of relevant publications, warranting investigations to decipher changes in other downstream caffeine metabolites.

Some studies have reported effect modification of estrogen and tobacco on the beneficial effects of caffeine on PD.^5,30,31^ Estrogen and caffeine are known to be competitively metabolized by CYP1A2, resulting in an inhibitory effect on caffeine metabolism.^40^ Conversely, tobacco has been shown to strongly induce the CYP1A2 enzyme, thereby increasing the metabolism of caffeine in smokers.^41^ Because metabolites of caffeine, such as paraxanthine and theophylline, are also A2AR antagonists and neuroprotectants, the net effect of perturbed caffeine metabolism is difficult to predict. In this study, we did not observe statistically significant effect modification by hormonal use and smoking. However, more marked trends toward a reduced risk of PD were observed among women who never used postmenopausal hormones with increasing coffee intake and among individuals who were both smokers and coffee drinkers. Our results might be hampered by limited power and results from literature on the estrogen-specific and smoking-specific modification of caffeine and PD are inconsistent. The interaction mechanism needs to be elucidated further in experimental studies.

As a widely consumed beverage, coffee has a discernible public health impact on PD prevention, though its effect size is relatively modest. However, the therapeutic effectiveness of caffeine and its metabolites for alleviating parkinsonian symptoms has been limited in current clinical trials.^9,10^ Together with our findings, these results suggest that caffeine may exert a neuroprotective effect during the prodromal phase, rather than after the onset of classical motor PD symptoms. Therefore, the administration of caffeine to individuals at high risk of PD, such as those with REM sleep behavior disorder, which is the strongest indicator of prodromal PD,^42^ could be a promising approach to stop or delay the disease deterioration. In parallel, it remains to be determined whether advocating for public intervention with promoting increased coffee consumption or caffeine supplements is appropriate due to the potential side effects of caffeine.

We acknowledged several limitations in our study. First, diet information and blood samples were collected on average 8 years before PD diagnosis. Coffee consumption behavior of participants can change due to an altered sense of taste and smell, which may occur up to 10 years before the onset of motor symptoms.^43^ Therefore, we cannot fully exclude that coffee consumption habits were secondary to PD-related symptoms. However, because consistent inverse associations between coffee/caffeine and PD risk were found as early as 12 years before disease diagnosis, residual reverse causality does not seem to be substantial. Second, the observed associations could be confounded by unmeasured factors. Genes *CYP1A2* and *ADORA2A*, which encode caffeine metabolism enzyme CYP1A2 and action target A2AR, respectively, might modify the protective effect of caffeine on PD.^44^ Environmental toxicants such as pesticides have been suggested to be associated with an increased risk of PD.^45^ However, to explain a 40% decrease in PD risk, any confounder must confer a risk ratio greater than 2.7 with both coffee consumption and PD risk.^46^ None of the known or suspected risk factors for PD demonstrate such strong associations. Thus, the chance that residual confounding fully explains our presented results is very limited. Third, coffee consumption assessed by dietary questionnaires might be subject to measurement error, particularly because of different coffee types and caffeine concentration in different countries. However, the dietary questionnaire has been validated in our cohort,^11^ and coffee intake was overall positively correlated with caffeine contents, although the extent varied across countries (eFigure 8, links.lww.com/WNL/D497). In addition, nondifferential measurement error would likely bias results toward the null, so the true coffee-PD associations could be stronger than the observed ones. Last, participants with missing data on coffee consumption and smoking status exhibited notable differences from the entire EPIC4PD population regarding demographics and recruitment countries (eTable 8). However, given the relatively small percentage of missing group (approximately 4%), the participants included in our study remained a representative sample of the EPIC4PD cohort.

In summary, our study validated the protective effect of caffeine on PD risk in a large prospective cohort and further confirmed the etiologic role of caffeine using biosamples before PD diagnosis in an untargeted exposomic framework. Our findings on the protective action of caffeine and its main metabolites provide insights into the etiology and prevention of PD.

## Appendix Authors

**Table:**

Name	Location	Contribution
Yujia Zhao, MSc	Institute for Risk Assessment Sciences, Utrecht University, the Netherlands	Drafting/revision of the article for content, including medical writing for content; major role in the acquisition of data; and analysis or interpretation of data
Yunjia Lai, PhD	Department of Environmental Health Sciences, Mailman School of Public Health, Columbia University, New York, NY	Drafting/revision of the article for content, including medical writing for content; major role in the acquisition of data
Hilde Konijnenberg, MSc	Institute for Risk Assessment Sciences, Utrecht University, the Netherlands	Drafting/revision of the article for content, including medical writing for content; analysis or interpretation of data
José María Huerta, PhD	Department of Epidemiology, Murcia Regional Health Council-IMIB, Murcia; CIBER Epidemiología y Salud Pública (CIBERESP), Madrid, Spain	Drafting/revision of the article for content, including medical writing for content
Ana Vinagre-Aragon, MD	Movement Disorders Unit, Department of Neurology, University Hospital Donostia; BioDonostia Health Research Institute, Neurodegenerative Diseases Area, San Sebastián, Spain	Drafting/revision of the article for content, including medical writing for content
Jara Anna Sabin	Division of Cancer Epidemiology, German Cancer Research Center (DKFZ), Heidelberg, Germany	Drafting/revision of the article for content, including medical writing for content
Johnni Hansen, PhD	Danish Cancer Institute, Danish Cancer Society, Copenhagen, Denmark	Drafting/revision of the article for content, including medical writing for content
Dafina Petrova, PhD	Escuela Andaluza de Salud Pública (EASP); Instituto de Investigación Biosanitaria-ibs.GRANADA, Granada; Centro de Investigación Biomédica en Red de Epidemiología y Salud Pública (CIBERESP), Madrid, Spain	Drafting/revision of the article for content, including medical writing for content
Carlotta Sacerdote, PhD	Unit of Cancer Epidemiology, Città della Salute e della Scienza University-Hospital, Turin, Italy	Drafting/revision of the article for content, including medical writing for content
Raul Zamora-Ros, PhD	Unit of Nutrition and Cancer, Cancer Epidemiology Research Programme, Catalan Institute of Oncology (ICO), Bellvitge Biomedical Research Institute (IDIBELL), Barcelona, Spain	Drafting/revision of the article for content, including medical writing for content
Valeria Pala, PhD	Epidemiology and Prevention Unit, Fondazione IRCCS Istituto Nazionale dei Tumori di Milano, Milan, Italy	Drafting/revision of the article for content, including medical writing for content
Alicia K. Heath, PhD	Department of Epidemiology and Biostatistics, School of Public Health, Imperial College London, United Kingdom	Drafting/revision of the article for content, including medical writing for content
Salvatore Panico, MD	School of Medicine, Federico II University, Naples, Italy	Drafting/revision of the article for content, including medical writing for content
Marcela Guevara, MD, PhD	CIBER Epidemiología y Salud Pública (CIBERESP), Madrid, Spain; de salud publica y laboral de Navarra, Pamplona, Spain; Navarra Institute for Health Research (IdiSNA), Pamplona, Spain	Drafting/revision of the article for content, including medical writing for content
Giovanna Masala, MD	Institute for Cancer Research, Prevention and Clinical Network (ISPRO), Florence, Italy	Drafting/revision of the article for content, including medical writing for content
Christina M. Lill, MD	Institute of Epidemiology and Social Medicine, University of Münster, Germany; Ageing Epidemiology Research Unit (AGE), School of Public Health, Imperial College London, United Kingdom	Drafting/revision of the article for content, including medical writing for content
Gary W. Miller, PhD	Department of Environmental Health Sciences, Mailman School of Public Health, Columbia University, New York, NY	Drafting/revision of the article for content, including medical writing for content; major role in the acquisition of data; and study concept or design
Susan Peters, PhD	Institute for Risk Assessment Sciences, Utrecht University, the Netherlands	Drafting/revision of the article for content, including medical writing for content; major role in the acquisition of data; and study concept or design
Roel Vermeulen, PhD	Institute for Risk Assessment Sciences, Utrecht University; University Medical Centre Utrecht, the Netherlands	Drafting/revision of the article for content, including medical writing for content; major role in the acquisition of data; and study concept or design

## Glossary

A2AR: adenosine 2A receptor
AAMU: 5-acetylamino-6-amino-3-methyluracil
AFMU: 5-acetylamino-6-formylamino-3-methyluracil
BMI: body mass index
EPIC: European Prospective Investigation into Cancer and Nutrition
ESI: electrospray ionization
HR: hazard ratio
IARC: International Agency for Research on Cancer
m/z: mass-to-charge ratio
OR: odds ratio
PD: Parkinson disease
PPF: population preventable fraction
RT: retention time
